# Using Small RNA-seq Data to Detect siRNA Duplexes Induced by Plant Viruses

**DOI:** 10.3390/genes8060163

**Published:** 2017-06-16

**Authors:** Xiaoran Niu, Yu Sun, Ze Chen, Rugang Li, Chellappan Padmanabhan, Jishou Ruan, Jan F. Kreuze, KaiShu Ling, ZhangJun Fei, Shan Gao

**Affiliations:** 1 College of Life Sciences, Nankai University, Tianjin 300071, China; niuxiaoran@mail.nankai.edu.cn (X.N.); sun_yu@mail.nankai.edu.cn (Y.S.); 2 State Key Laboratory of Veterinary Etiological Biology and Key Laboratory of Veterinary Parasitology of Gansu Province, Lanzhou Veterinary Research Institute, Chinese Academy of Agricultural Science, Lanzhou 730046, Gansu, China; chenze@caas.cn; 3 U.S. Department of Agriculture-Agricultural Research Service, U.S. Vegetable Laboratory, Charleston, SC 29414, USA; rugangli@yahoo.com (R.L.); Chellappan.padmanabhan@ars.usda.gov (C.P.); Kai.ling@ars.usda.gov (K.L.); 4 School of Mathematical Sciences, Nankai University, Tianjin 300071, China; jsruan@nankai.edu.cn; 5 International Potato Center (CIP), Apartado 1558, Lima 12, Peru; j.kreuze@cgiar.org; 6 Boyce Thompson Institute, Cornell University, Ithaca, NY 14853, USA

**Keywords:** siRNA duplex, RNAi, plant virus, small RNA-seq, virus detection

## Abstract

Small interfering RNA (siRNA) duplexes are short (usually 21 to 24 bp) double-stranded RNAs (dsRNAs) with several overhanging nucleotides at both 5′- and 3′-ends. It has been found that siRNA duplexes bind the RNA-induced silencing complex (RISC) and cleave the sense strands with endonucleases. In this study, for the first time, we detected siRNA duplexes induced by plant viruses on a large scale using next-generation sequencing (NGS) data. In addition, we used the detected 21 nucleotide (nt) siRNA duplexes with 2 nt overhangs to construct a dataset for future data mining. The analytical results of the features in the detected siRNA duplexes were consistent with those from previous studies. The investigation of siRNA duplexes is useful for a better understanding of the RNA interference (RNAi) mechanism. It can also help to improve the virus detection based on the small RNA sequencing (sRNA-seq) technologies and to rationally design siRNAs for RNAi experiments.

## 1. Introduction

RNA interference (RNAi) is a cytoplasmic cell surveillance system that recognizes double stranded RNAs (dsRNAs) and specifically destroys single and double stranded RNA molecules homologous to the dsRNA inducers, using small interfering RNAs (siRNAs) as guides [1]. The abundant siRNAs accumulated during the RNAi process can be captured by the small RNA sequencing (sRNA-seq) technology that has been used for virus detection in plants [2,3,4] and invertebrates [5,6]. However, the detection of viruses in somatic mammalian cells using sRNA-seq is hampered by the presence of a number of dsRNA-triggered nonspecific responses such as the type I interferon (IFN) synthesis [7], although it is well known that antiviral RNAi functions in mammalian germ cells and embryonic stem cells (ESCs), as well as some carcinoma cell lines [8]. In 2016, Wang et al. first used big data from the National Center for Biotechnology Information (NCBI) Sequence Read Archive (SRA) database to prove that sRNA-seq can be used to detect and identify human viruses [1], but the detection results were not as good as those of plant viruses. The genome coverages and average depths of detected mammal viruses were much lower than those of detected plant viruses. The study of RNA fragments related to RNAi could help to find some featured RNA fragments to improve the virus detection in mammals.

One important class of RNA fragments related to RNAi is siRNA duplexes, which contain perfectly base-paired regions with 2 nucleotide (nt) 3′-end overhangs. Previous studies have shown that siRNA duplexes of 21–23 nt are the sequence-specific mediators of RNAi and post-transcriptional gene silencing (PTGS) [9]. The activity of siRNA duplexes in RNAi is largely dependent on their binding ability to the RNA-induced silencing complex (RISC). Binding of siRNA duplexes to RISC is followed by unwinding and cleavage of the sense strands in siRNA duplexes with endonucleases. RISC then uses the remaining antisense strands to target mRNAs and initiate transcriptional silencing. It has also been reported that siRNA duplexes with 3′-end overhangs of 2 or 3 nt more efficiently result in RNA degradation compared with blunt-ended duplexes, and the most potent siRNA duplexes are 21 nt long [9]. Although previous studies have revealed some biological principles from the generation of siRNA duplexes to their silencing effects, these results are mainly based on the data from the conventional technologies (e.g., Northern blot) and thus cannot provide information that is as comprehensive as those based on the next-generation sequencing (NGS) data.

Since plant RNAi produces abundant siRNA duplexes and the mechanism of plant RNAi is comparatively clear, in this study we detected siRNA duplexes induced by plant viruses and analyzed their features. As far as we know, this is the first time siRNA duplexes have been detected and analyzed on a large scale using NGS data. This study aims to provide useful information for a better understanding of the RNAi mechanism. The analysis of siRNA duplexes can be used to improve the virus detection using sRNA-seq data and to rationally design siRNAs for RNAi experiments [10].

## 2. Materials and Methods

Fourteen complete viral genomes including 17 nucleic acid sequences under the NCBI GenBank accession numbers JQ314457 [3], JQ314458 [3], JQ314459 [3], JQ314460 [3], JQ314461 [3], JQ314462 [3], JQ314463 [3], KT438549 [11], KT634055 [12], KT810183 [13], KM504246 [14], KM504247 [14], KM504248 [14], KR094068 [15], KP772568 [16], KP223323 [17] and KP223324 [17] were used in this study. These 14 viruses had been detected from sRNA-seq data using VirusDetect [4] and their genome sequences were confirmed using reverse transcription PCR (RT-PCR) with Sanger sequencing. Finally, 5′ and 3′ rapid amplification of cDNA ends (5′ RACE-PCR and 3′ RACE-PCR) were used to obtain the complete sequences.

The cleaning and quality control of sRNA-seq data were conducted using the pipeline Fastq_clean [18] that has been optimized to clean the raw reads from Illumina platforms [19,20,21,22,23,24,25]. Using the software Bowtie v.0.12.7 [26] with one mismatch, we aligned all the cleaned sRNA-seq reads to the 17 viral genome sequences and calculated the average depths and the genome coverages. The average depth is calculated as the total number of nucleotides of the aligned reads divided by the read-covered positions on the reference genome. The genome coverage represents the proportion of read-covered positions against the genome length. The *x*–nt duplex (*x* represents the duplex length) proportion is calculated as the read count of *x*–nt siRNA duplexes with 2 nt overhangs divided by the count of viral reads. The program duplexfinder was developed to detect siRNA duplexes [27]. Statistics and plotting were conducted using the software R v2.15.3 with the Bioconductor packages [28].

## 3. Results and Discussion

All 17 viral genome sequences had genome coverages of more than 99% and average depths (Section 2) varied from 13.61 to 4515.53 (Table 1). Seven of the 17 viral sequences (KM504246, KM504247, KM504248, KR094068, KP772568, KP223323, and KP223324) had average depths above 2000, which were significantly higher than the average depths of the other ten viral sequences. However, the sRNA-seq data that contained the seven viral sequences with higher average depths did not have higher sequencing depths than the sRNA-seq data that contained the other ten viral sequences with lower average depths. This suggested that the sequencing depth determines the genome coverage and the average depth for virus detection, but it cannot yield additional information over a threshold.

**Figure 1:**
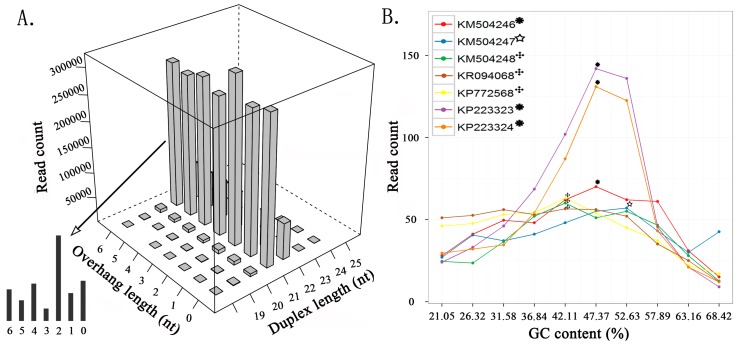
Read-count distribution of small interfering RNA (siRNA) duplexes. (**A**) The read count of siRNA duplexes varies with the duplex length and the overhang length, using KP772568 as an example. (**B**) The median of read counts varies with the internal GC content using 21 nt siRNA duplexes with 2 nt overhangs from seven viral sequences.

To reduce the statistical bias, we used seven nucleic acid sequences with higher average depths from four viruses to detect and analyze siRNA duplexes. The duplex lengths from 15 to 50 nt and the overhang lengths from 0 to 6 nt were used as parameters to count the duplex reads in the sRNA-seq data (Figure 1A). The results showed that the duplex length was the principal factor to determine the read count. The 21 nt siRNA duplexes with 2 nt overhangs were the most abundant duplexes, followed by the 22 nt siRNA duplexes with 2 nt overhangs. This finding is consistent with that in a previous study, which proved that 21 nt siRNA duplexes with 2 nt overhangs were the most efficient triggers of mRNA degradation in *Drosophila melanogaster* embryo lysates [9]. Among the seven sequences, KP223323 had the highest 21 and 22 nt duplex proportion (Section 2) of 47.06% (630,858/1,340,402) and 27.68% (371,047/1,340,402), respectively, which were very close to the highest duplex proportion 45% and 28% estimated in the *Drosophila* in vitro system [29]. These results suggested that plants and invertebrates could share common mechanisms in the RNAi process.

**Figure 2:**
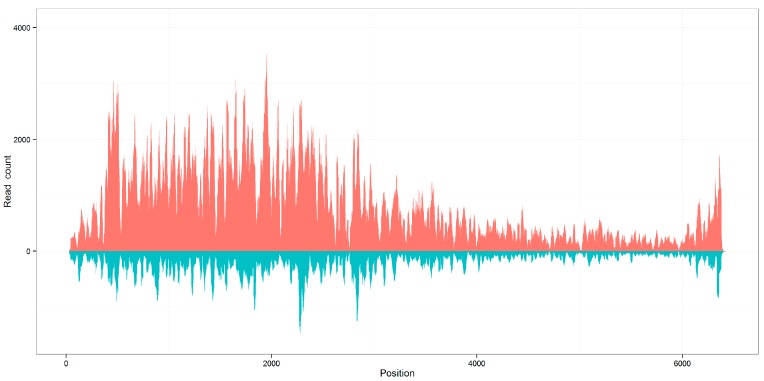
Distribution of viral reads along the reference genomes. The genome coverages of seven viral sequences calculated using aligned 21 nt siRNA duplexes with 2 nt overhangs are close to the genome coverages calculated using all aligned reads, using KP772568 as an example. The results of all 14 sequences can be seen in Supplementary File 1.

In this study, we also found genome coverages of the seven viral sequences calculated using aligned 21 nt siRNA duplexes with 2 nt overhangs were close to the genome coverages calculated using all aligned reads (Supplementary File 1). Identical to the distribution of all aligned reads, the distribution of 21 nt siRNA duplexes with 2 nt overhangs along the plant viral genomes was also not even (Figure 2). The 21 nt duplex proportions and the average depths of the seven viral sequences were above 20% and 100×, respectively. From Table 1, it can be understood that the count of viral reads, the average depth, and the duplex proportion had positive correlations. This finding suggested that the efficient virus detection required the capture of adequate 21 nt siRNA duplexes with 2 nt overhangs and these duplexes could play a more important role in the plant RNAi process.

Based on the detection results from the seven viral sequences, we constructed a dataset including 20,415 pairs of 21 nt siRNA duplexes with 2 nt overhangs for further analysis (Supplementary File 2). Using this dataset, we found that the read-count distribution of 21 nt siRNA duplexes was associated with GC contents of 19 nt internal base pairs. The highest medians of read counts in KM504248, KR094068, and KP772568 were associated with the internal GC content of 42.11% (8/19), and the highest medians in KM504246, KP223323 and KP223324 and the highest median in KM504247 were associated with internal GC contents of 47.37% (9/19) and 52.63% (10/19), respectively (Figure 1B). Since previous studies have shown that siRNA duplexes with internal GC contents of 36.84%, 42.11%, 47.37%, and 52.63% resulted in the best RNAi effects in mammals, our results suggested that 21 nt siRNA duplexes with 2 nt overhangs and internal GC contents of 42.11% and 47.37% could be used as the criteria to design siRNAs for gene targeting. Additionally, previous studies have shown that the 2 nt 3′ overhangs are critical to RNAi function, and the most efficient siRNA duplexes have the overhang quadmer type NN/UG, NN/UU, NN/TdG, and NN/TT (dG represents 2′-deoxyguanosine and N represents any nucleotide) [9]. In this study, we investigated the abundance of 256 possible quadmer types (NN/NN) in the siRNA duplex dataset. Among the 16 appeared quadmer types with internal GC contents of 42.11% or 47.37%, CC/CC was the most abundant type and AA/AA was the least abundant type.

**Figure 3:**
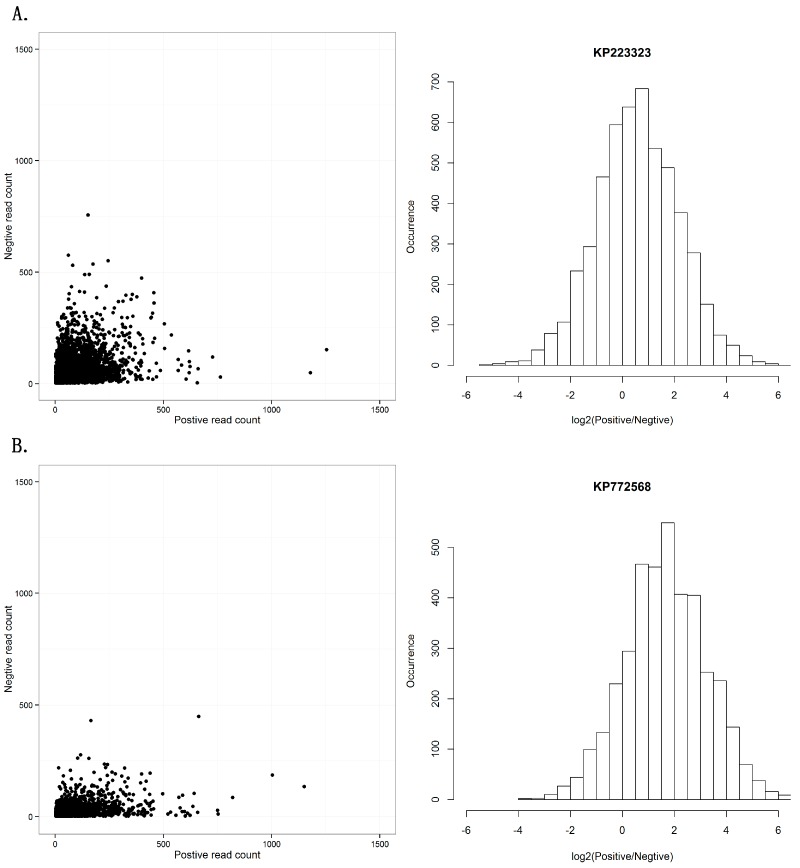
Positive and negative-strands of siRNA duplexes. (**A**) This pattern shows symmetric read-count distribution of positive and negative strands in 21 nt siRNA duplexes with 2 nt overhangs, using KP223323 as an example. (**B**) This pattern shows read-count distribution biased to positive strands in 21 nt siRNA duplexes with 2 nt overhangs, using KP772568 as an example. The results of all seven sequences can be seen in Supplementary File 3.

Another controversial topic in RNAi studies is whether the RISC contains single- or double-stranded siRNAs. Previous studies have introduced a debate on the symmetry between sense and antisense strands of siRNAs [29]. Using the dataset of the seven viral sequences, we investigated the distribution of viral reads aligned on the positive and negative strands (Supplementary File 3). The seven viral sequences showed two different patterns in the distribution of positive- and negative-stranded counts. One pattern from the sequences KM504246, KM504247, KM504248, KP223323, and KP223324 had symmetric read-count distribution of positive and negative strands (Figure 3A), while the other pattern from the sequences KR094068 and KP772568 had a read-count distribution biased to positive strands (Figure 3B). Although the two patterns were different, this result still confirmed our previous study that positive single-stranded RNA viruses usually had siRNAs from both strands and double-stranded DNA viruses had siRNAs from sense strands.

## Figures and Tables

**Table 1:** Viral sequences used in this study.

ID	Description	Viral Read	Depth (bp)	Coverage	Proportion
JQ314457	Pepino mosaic virus strain EU_CAHN8, complete genome	8349	28.39	98.27%	0.57%
JQ314458	Pepino mosaic virus strain US1_CAHN8, complete genome	11,651	39.28	99.50%	0.89%
JQ314459	Pepino mosaic virus strain EU_EF09_58, complete genome	90,569	298.61	99.95%	6.97%
JQ314460	Pepino mosaic virus strain US1_EF09_58, complete genome	21,374	71.95	99.84%	0.51%
JQ314461	Pepino mosaic virus strain EU_EF09_60, complete genome	36,002	120.81	99.94%	2.01%
JQ314462	Pepino mosaic virus strain US1_EF09_60, complete genome	47,776	160.46	99.92%	4.60%
JQ314463	Tomato necrotic stunt virus strain MX9354, complete genome	207,553	439.85	100.00%	12.72%
KT438549	Southern tomato virus isolate CN-12, complete genome	4508	27.93	99.10%	0.64%
KT634055	Southern tomato virus BD-13, complete genome	2161	13.61	100.00%	0.00%
KT810183	Tomato mottle mosaic virus isolate NY-13, complete genome	89,061	292.25	100.00%	26.75%
KM504246 *	Tobacco streak virus isolate FL13-07 segment RNA1, complete sequence	1,141,881	4515.53	100.00%	21.77%
KM504247 *	Tobacco streak virus isolate FL13-07 segment RNA2, complete sequence	635,836	3864.01	100.00%	21.23%
KM504248 *	Tobacco streak virus isolate FL13-07 segment RNA3, complete sequence	499,308	4252.48	100.00%	21.67%
KR094068 *	Melon necrotic spot virus isolate ABCA13-01, complete genome	969,376	4380.00	100.00%	39.32%
KP772568 *	Cucumber green mottle mosaic virus isolate ABCA13-01, complete genome	635,907	2113.00	100.00%	33.50%
KP223323 *	Squash mosaic virus segment RNA-1, complete sequence	1,340,402	4454.95	100.00%	47.06%
KP223324 *	Squash mosaic virus segment RNA-2, complete sequence	638,694	3808.92	100.00%	48.22%

ID are the GenBank accession numbers. Viral read represents the number of reads that can be aligned to this viral sequence using Bowtie software, allowing one mismatch. Depth (average depth) is calculated as the total number of nucleotides of the aligned reads divided by the read-covered positions on the reference genome. Coverage (genome coverage) represents the proportion of read-covered positions against the genome length. Proportion (21 nt duplex proportion) is calculated as the read count of 21 nt siRNA duplexes with 2 nt overhangs divided by the count of viral reads. * Seven sequences with higher average depths from four viruses were used to detect and analyze siRNA duplexes.

## Supplementary Materials

The following are available online at www.mdpi.com/2073-4425/8/6/163/s1. Supplementary File 1: Distribution of viral reads along the reference genome, Supplementary File 2: All the 21-nt siRNA duplexes with 2-nt overhangs, Supplementary File 3: Positive and negative-strands of siRNA duplexes.
